# Intraoperative Frozen Section Analysis for Margin Status in Breast Conserving Therapy: a Retrospective 6-Year Experience at a Tertiary Centre in North East India

**DOI:** 10.1007/s13193-025-02301-z

**Published:** 2025-05-06

**Authors:** Dibyajyoti Deka, Clara Atieno Odhiambo, Abhijit Talukdar, B. B. Borthakur, Pompi Daimari Buragohain, Deep Jyoti Kalita, Gaurav Das, Shivaji Sharma

**Affiliations:** 1 Institute of Department of Atomic Energy, Bhubaneswar Borooah Cancer Institute (Tata Merorial Centre), Guwahati, Assam India; 2 Office of HOD, Dept of Surgical Oncology, Dr B. Borooah Cancer Institute, Gopinath Nagar, A K Azad Road, Guwahati, 781016 India

**Keywords:** Frozen section analysis, Breast conserving surgery, Accuracy

## Abstract

Breast cancer is the commonest cancer among Indian women as it is globally. Margin status post lumpectomy remains an important predictor of local recurrence after breast conserving surgery. We set out to investigate the positive predictive value of intra operative frozen section analysis in a tertiary cancer center in North East India. Retrospective data from all women who underwent breast conserving Surgery (BCS) from 2017 to 2022 was included. Frozen section analysis reports were compared against final pathology reports. Comparison was in regard to margin status. Two hundred ten women underwent BCT, and mean age was 49.5 years. The sensitivity and specificity of frozen section was 92.5% (86.2–95.64% 95% CI) and 99.8% (62.23–99.9% 95% CI) respectively. The PPV and NPV was 94.8% (87.09–99.86% 95% CI) and 99.8% (95.53–99.9% 95 CI). Our analysis showed an accuracy of 99.63% (95.22–99.96%, 95% CI). We concluded that frozen section analysis is accurate and has a high positive predictive value and negative predictive value for margin status in breast conserving surgery.

## Introduction

The most common cancer among Indian women is now breast cancer [1]. The incidence of breast cancer in India is rising, and this rise is being seen in younger women. Globally, as reported by the Global Cancer Observatory in 2020, about 2,261,419 new cases of breast cancer were diagnosed [2]. Deaths from breast cancer were 684,996. GLOBOCAN predicts that in the next twenty years the burden of breast cancer will increase to over 3 million new cases and 1 million deaths per year (GLOBOCAN) [3].

Breast Conservation Therapy (BCT) refers to limited breast resection with lymph node dissection typically followed by breast irradiation. In early breast cancer, BCT has become the mainstay of treatment. BCT has also been shown to have comparable overall survival with mastectomy in the control of breast cancer [4]. Margin status post lumpectomy remains an important predictor of local recurrence after breast conservation surgery (BCS) and radiation therapy [5]. For a subset of women with large tumour’s that would have otherwise undergone mastectomy, neoadjuvant treatment with chemotherapy can cause some tumour’s to shrink, thereby making such tumour’s amenable to BCS. Randomized clinical trials (RCTs) have shown the same survival rates between groups receiving perioperative chemotherapy (NSABP B18, EORTC 10902). With the availability of oncoplastic surgery techniques, indications for BCS are extending; moreover, studies have shown the safety of oncoplastic surgery after receiving neoadjuvant chemotherapy [6].

Frozen section is a rapid diagnostic procedure performed on tissues obtained intraoperatively [7]. It is among the many methods of margin assessment used. As an intraoperative margin assessment technique, it has a high diagnostic accuracy [8]. It has been shown to significantly reduce reoperation rates [9]. To execute intraoperative frozen section analysis smoothly, there must be a dedicated collaboration between the surgeon, pathologist, and radiologist. Reoperation goes hand in hand with increased surgical costs, patient anxiety, dissatisfaction, and poorer cosmetic outcomes [10]. Undoubtedly, the gold standard for margin assessment is paraffin sections of inked surgical margins.

In our center, we decided to review our experience with intraoperative frozen section analysis for determining margin status in patients with invasive breast cancer undergoing conservation, aiming to evaluate its accuracy.

## Methodology

A retrospective analysis was performed at Dr. B Borooah Cancer Institute, Guwahati. Data of 210 women who had undergone breast-conserving surgery were retrieved. A diagnosis of invasive breast cancer was made by core needle biopsy. The study covered women who had been treated at the institution from January 2017 to December 2022. The study was approved by the ethical committee of the authors institute.

The study determined the following parameters: age of the patients, biological type of cancer, histological grade, receptor status (ER, PR, and HER2 NEU), presence of DCIS, percentage of patients who had received neoadjuvant chemotherapy, Miller Payne regression score for patients who had received neoadjuvant chemotherapy, axillary treatment, the presence or absence of oncoplastic procedures, and postoperative radiation.

### Surgery

Surgery was carried out in our institution for all the patients, and they all fulfilled accepted criteria for breast-conserving surgery. Patients who had received breast-conserving surgery from other institutions apart from the authors were excluded from the study. Similarly, patients whose final histopathology report had benign disease were excluded from analysis. Patients who had not been followed up for a minimum of one year after surgery were excluded from the study. Informed consent was obtained for breast-conserving surgery as well as mastectomy if the need for the latter arose. Surgery was either performed upfront for early breast cancer or after neoadjuvant chemotherapy. Patients who were deemed not fit for upfront surgery had tattooing of tumour margins with injectable methylene blue intradermally as an outpatient procedure. Such patients proceeded to have their neoadjuvant treatment, and then reassessment was done for surgery. Resections were not done according to the pre-chemo tattooed marking. Actually at that time period (2017–2022), we did not have a facility for placement of markers before starting chemotherapy. We did tattoo marking because we had a few cases earlier where there was complete clinical response, so the patient had to undergo mastectomy due to lack of reference site. Tattoos were faintly visible in most cases at the end of chemotherapy. Tattooed skin is not excised. Tattooed sites come to action only if the patient has a complete clinical response and no lesion is seen even on ultrasound. Then we excised the marked part. Otherwise, in patients with partial response, we take the new margin around the shrunken tumour. The specimen was oriented routinely as short superior and long lateral for superior and lateral margins, respectively, before complete excision, and once done, handed over for frozen section analysis as well as paraffin section. For a group of patients who had a clinical complete response, pre-operative image-guided wire localization was done. Wire localized lesions were subjected to specimen mammography intraoperatively after complete excision. All the specimens of BCT were not sent for specimen mammography routinely. Specimen mammography was done only for specimens where the tumour was not identifiable in pre-surgery imaging. The operating surgeon had to confirm that the tip of the wire is in the centre of the excised specimen so that there is an adequate margin all around it before handing it over to the pathologists. Metallic clips were placed on the lumpectomy bed for adjuvant radiation boost purposes. The axilla was staged by either axillary dissection or sentinel lymph node biopsy.

### Pathological Evaluation

**Fig. 1:**
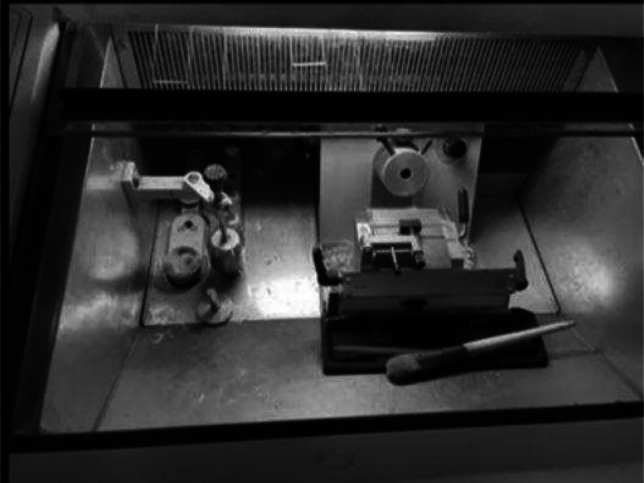
Cryostat with freezing chamber and cryotome

**Fig. 2:**
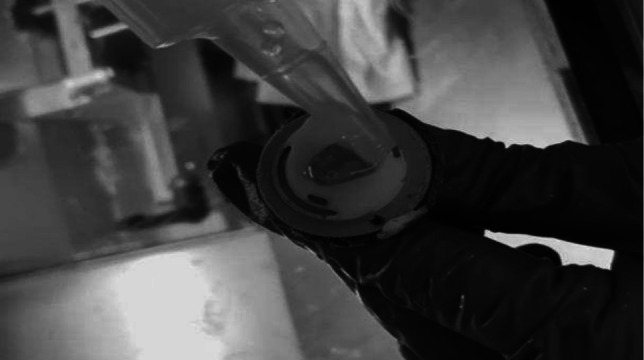
Embedding medium is applied on LN placed on specimen dise

**Fig. 3:**
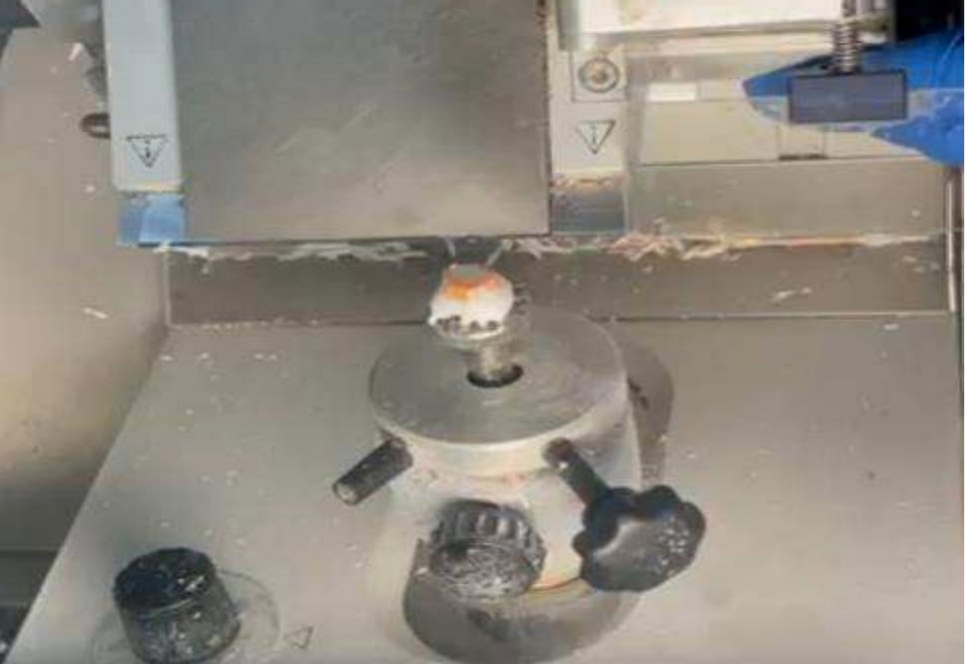
Once frozen, LN is placed on specimen head

**Fig. 4:**
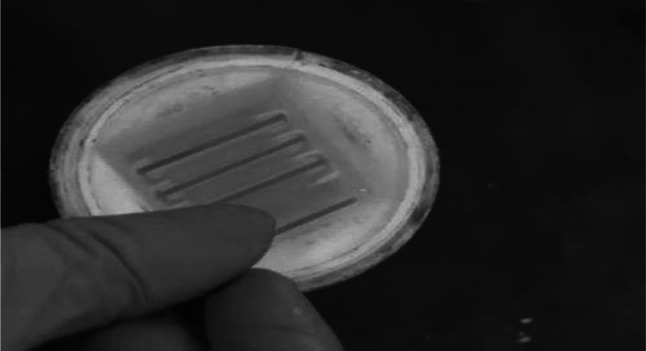
Fixed in alcohol

**Fig. 5:**
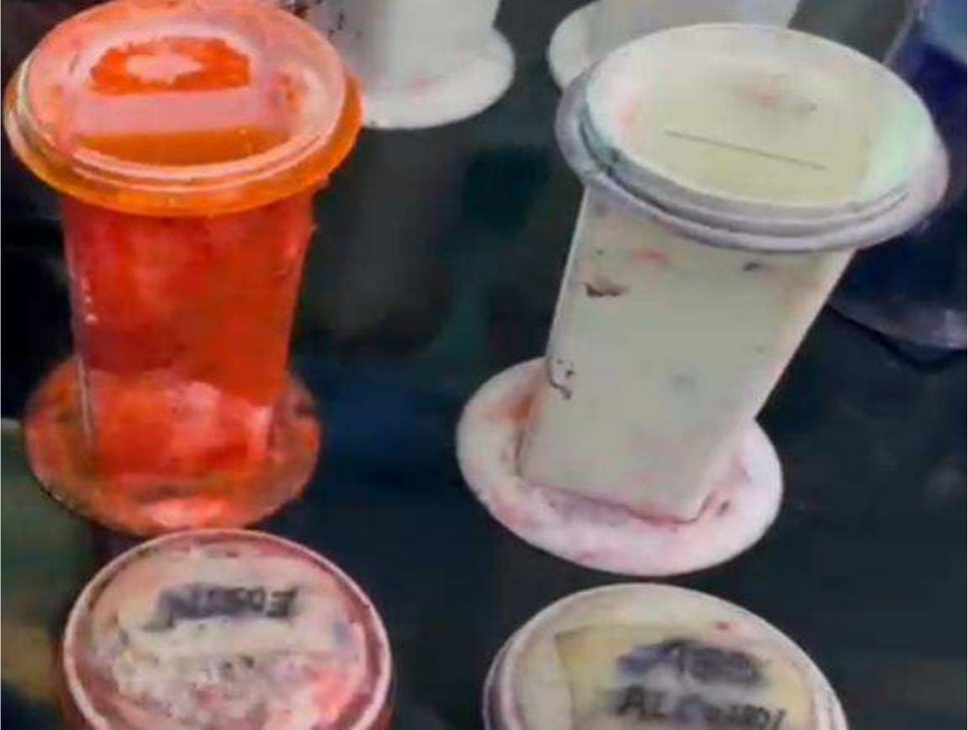
Stained serially in H and E

**Fig. 6:**
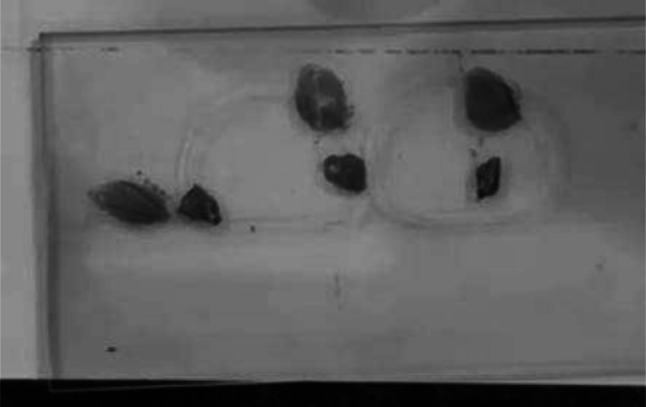
Slides ready to be examined under microscope

The specimen was immersed in normal saline and transported to the pathology laboratory for frozen section analysis. Once in the pathology lab, the tissue was bisected or trisected depending on the size of the tissue; this applied to sentinel nodes. The lumpectomy specimen was first grossed and inked by the pathologist, then placed on a thin layer of cryo-compound in the cryostat machine, then cooled at − 24˚C or more. The specimen was then mounted onto the microtome and trimmed so as to achieve a full face section of the specimen. Once this was achieved, the micrometre was set to the standard section value of 5 µm. The frozen tissue was sectioned, and a glass slide was lowered onto the section. The section automatically adhered to the glass slide. It was then stained with haematoxylin and eosin and read under a microscope. (Figs. 1, 2, 3, 4, 5, and 6). Results were recorded as positive, negative, or suspicious with respect to different margins.

A positive result was defined as ink on the tumour margin, whereas a negative result did not have the tumour touching the inked margin. When the tumour was very close to the inked margin, the distance was measured and indicated in millimetres. A suspicious result was given when atypical cells were seen but not identifiable as invasive carcinoma. This information would be conveyed to the operating surgeon over the phone. In case margins came back positive, there would be a re-excision of the margin and the process of frozen section repeated. If a margin was reported positive for the second time, the ultimate cosmetic result would be weighed against re-excision. If a poor cosmetic outcome would result, the next of kin would be informed in real time, and the surgery would be changed to mastectomy.

The final report described in detail the initial frozen section analysis report on margin status and a thorough narrative on the final tumour seen, including the size of the tumour, histological grade, presence of lymph vascular invasion, presence of perineural invasion, presence of DCIS, distance of the tumour from resected margins, closest margin to the tumour, status of revised margins, receptor status, pathological response grading for patients who had received neoadjuvant treatment, number of lymph nodes retrieved, and positive or negative nodes with and without extra nodal extension.

### Follow-up

All patients received adjuvant chemotherapy, some as completion. The latter had received neoadjuvant treatment. Standard adjuvant treatment comprised taxane- and anthracycline-based chemotherapy. Targeted therapy was offered to those who were HER2 NEU positive. Hormonal therapy was added after chemotherapy and radiotherapy for hormone-positive cancers. Radiotherapy was administered by a linear accelerator to the breast to a maximum of 40.05 Gy in 15 fractions, followed by 12.5 in 5 fractions. After completing treatment, patients were followed up every 3 months for the first 2 years. Then followed by 6 monthly for the next 3 years. All patients usually had annual mammography unless indicated for early mammography.

### Statistical Analysis

All statistical analysis was carried out using the SPSS 11.5 statistical package program. Continuous variables were analyzed by using the mean and median. Categorical variables were measured in proportions and percentages. The effectiveness of intraoperative frozen section analysis was measured by sensitivity and specificity as well as the positive and negative predictive values.

## Results

Between January 2017 and December 2022, two hundred ten women had undergone breast conserving surgery at the Institute. The mean age of the women undergoing breast conserving surgery was 49.5 years (Tables 1 and 2).

**Table 1:** Patient characteristics

Characteristic	N = 210	n (%)
Age, mean age (years)	49.5 ± 12.3	-
Tumor		
Ductal hyperplasia	1	0.5%
Fibroadenoma	2	1%
Paget’s	1	0.5%
Phyllodes	2	1%
No malignancy	16	7.9%
DCIS	11	5.4%
LCIS	1	0.5%
Invasive breast cancer	162	77.2%
Metaplastic	2	1%
Mucinous	4	2%
Papillary	6	3%
Molecular Subtypes		
Luminal A	67	31.9%
Luminal B	48	22.9%
HER 2 enriched	17	8.1%
Basal like	32	15.2%
Not traceable	46	21.95
Neo adjuvant Chemotherapy		
Had Neo adjuvant Chemotherapy	32	15.8%
Had no Neo adjuvant Chemotherapy	178	84.2%
Breast Reconstruction		
Had Breast Reconstruction	9	4.4%
Had no Breast Reconstruction	201	95.6%

**Table 2:** Details of total examined margin

Total margin examined	1092
Positive margin	**39**
True positive	**37**
False positive	**2**
False negative	**3**
True negative	**1050**

The sensitivity and specificity of intraoperative frozen section analysis in determining margin status was as depicted in Table 3.

**Table 3:** Sensitivity and specificity of intraoperative frozen section analysis

Statistic	Value	95% CI
Sensitivity	92.5%	86.2 to 95.64%
Specificity	99.8%	62.23 to 99.9%
Positive Predictive Value	94.8%	87.09 to 99.86%
Negative Predictive Value	99.8%	92.53 to 99.9%
Accuracy	99.63%	95.22 to 99.96%

Seventy-one (33.8%) patients were premenopausal and perimenopausal. 0.7%, 2.3%, and 21.9% were grade 1, grade 2, and grade 3, respectively. Nine out of 210 (18.57%) patients required re-excision to attain negative margins.

Thirty-two (15.6%) patients received neoadjuvant chemotherapy, and 12 out of these achieved a pathological complete response on permanent section. Five out of the 12 who achieved a pathological complete response (pCR) on permanent section had their frozen section report as suspicious for residual with no definite opinion on margin. Here 12 is the number of patients who had pCR as per the final HPE report. But in these 12 patients, when freezing was done intraoperatively in 5 cases, pathologists were not sure whether the tumour had disappeared or not. We did margin revision in these cases, but in all 5 cases, the, the final HPE was reported as negative for residual tumour in the main specimen itself.

A total of 1092 margins were examined, including 39 revised margins. These margins were revised as they were marked as positive or suspicious. Out of the total 1092 margins (primary surgery + revised margin), only 3 margins had false negative reporting during the frozen section. In this small percentage (0.29%), which were reported as negative margins, the final HPE was a positive margin.

Two patients with persistent positive margins on frozen section underwent mastectomy during initial surgery.

Three patients with reported negative margins on frozen section turned out to have positive margins on permanent section and had mastectomy within four weeks of the first surgery.

Twenty-two patients were treated with sentinel lymph node biopsy, and 6 out of these had their sentinel nodes positive for malignancy. The rest were reported as reactive nodes. A formal axillary dissection was carried out for these 6 patients. In the the permanent section, 1 patient had reactive nodes (non-malignant) identified. The rest had evidence of malignancy in axillary nodes.

A total of 167 (79.5%) patients received adjuvant radiation; 42 (20%) did not receive any radiation and in this 42 patients radiation was not omitted. They were RT defaulters. Of the 167 who were irradiated, 5 developed recurrences, 4 distant metastases, and 4 died. Among the patients who did not receive adjuvant radiation, there were no reported recurrences or distant metastases, but one patient died, and the cause of death was not ascertained.

In this study population, follow-up ranged from 6 to 120 months. During this period, 5 (2.4%) patients died; two of these were among the four reported to have developed distant metastases. It was not ascertained whether the remaining three died of breast cancer-related issues.

Four (1.9%) patients developed distant metastasis; one to the spine, two to the liver and one to both the liver and lungs, as shown in Table 4.

**Table 4:** Showing patients who developed distant metastasis

Age	Receptor status	Margin status	Axilla nodal yield	LVI/PNI** status	NACT*** status
31	TNBC^*^	Negative	13 all reactive	-/-	post NACT
58	ER/PR/HER 2 +	close margin (0.1 cm)	15/30 with metastases	-/-	NACT naïve
59	Not traceble	close margin (0.2 cm)	18 all reactive	-/-	NACT naïve
59	ER/PR - HER 2 +	Negative	1/7 with metastases	-/+	post NACT
35	ER/PR + HER 2 -	Negative	6/13 with metastases	±	NACT naïve

^*^TNBC triple negative breast cancer

^**^LVI/PNI lymphovascular invasion/perineural invasion

^***^NACT neo adjuvant chemotherapy

Table 5 shows 4 (1.9%) patients who developed loco regional recurrence. Two out of these defaulted on adjuvant treatment.

**Table 5:** Showing patients who developed loco regional recurrence

Age	Receptor status	Margin status	Axilla nodal yield	NACT status	Site of recurrence
57	TNBC	Negative	12 all reactive	post NACT	Nodes
31	TNBC	Negative	5/15 with metastases	NACT naïve	Ipsilateral breast
40	TNBC	Negative	0	post NACT	Contralateral breast *
73	TNBC	Negative	5/19 with metastases	NACT naïve	Ipsilateral breast

^*^Patient was treated for hormone positive breast cancer eight years ago. Present tumor was triple negative and recurred in the opposite breast

## Discussion

This study found that intraoperative frozen section analysis for margin status was highly sensitive and specific. The positive predictive value stood at 99.17% with an accuracy of 82.24% [11–13].

In a meta-analysis by Garcia et al., he determined an accuracy of frozen section analysis at 0.98. Sensitivity and specificity in the same study were 0.81 and 0.97, respectively [14]. Despite the large sample size, these results were comparable to ours, where we determined a sensitivity of 0.82 and a specificity of 0.85. Intraoperative frozen section analysis is reliable and has been reported to reduce reoperation rates [15, 16].

Many studies have shown the sensitivity of frozen section analysis in the ranges of 84.6–97.9%. Likewise, reported specificities are between 94 and 100%. In our study both sensitivity and specificity of intraoperative frozen section analysis were near the lower range reported in the literature [17].

Whereas intraoperative frozen section analysis is time-consuming and increases overall operation time, when compared to repeated surgeries and associated morbidities, the benefits have been seen to outweigh the risk. Worse still, reoperation delays adjuvant treatment. The reoperation rate in this study of 1.5% compared favourably with a study conducted by Farouk et al. in Egypt [18]. Out of 219 patients evaluated over a 7-year period, 3 patients, representing 1.4%, required reoperation (mastectomy) [19].

Discordant rates have also been shown to occur. Literature quotes about 1.4–12.95% occurrence. This study showed a low rate of positive margins on final formalin-fixed paraffin-embedded sections. We had 3 patients, representing 1.5% of those whose initial frozen section analysis showed negative margins [18]. Similarly, Althoubaity et al. studied 110 women and found 5.5% positive margins on permanent histopathology [20].

Since intraoperative frozen section analysis requires that information regarding margin status be availed before closure or reconstruction of the lumpectomy cavity, chances are that margin revision may be performed and tissue submitted for frozen section analysis of the revised margin. In our study, 18.57% required margin revision intraoperatively to attain negative margins. A study by T. P. Olson et al. hypothesised that lumpectomy with intraoperative frozen section analysis of cavity biopsies would prevent re-excisions and result in low recurrence rates. His study showed a low rate of local recurrence of 2.1% [21]. These results were comparable to those in our study where 4 patients, representing 1.9%, developed local recurrence [22].

The majority of the patients received adjuvant radiation therapy. Adjuvant radiation has been shown to reduce local recurrence after breast conservation surgery [23]. In our study, half of the patients who developed local recurrence defaulted in their adjuvant radiation treatment.

## Conclusion

Intraoperative frozen section analysis is an accurate, quick and reliable method for establishing margin status in breast conservation surgery. With its use comes increased costs in terms of additional diagnostic requirements as well as prolonged operative time. Notwithstanding, it is efficacious in that it greatly reduces the rates of repeat surgeries. Through the evaluation of sentinel lymph nodes intraoperatively for metastasis, it guides the surgeon in avoiding overtreatment in regard to axillary lymph node dissection and vice versa. A good and robust, available multidisciplinary team comprising surgeons, pathologists, radiologists, and their respective technicians allows for the smooth execution of the procedure. Frequent audits on the quality of frozen section analysed specimens help to improve the overall reliability.

### Limitation of the Study

It was a retrospective study, so we had to depend on electronic medical records for data collection. The follow-up time period was shorter to comment on actual recurrences post BCS.

## Data Availability

The data underlying this study are available from the corresponding author upon reasonable request.
